# Thymoquinone-Enriched Naringenin-Loaded Nanostructured Lipid Carrier for Brain Delivery via Nasal Route: In Vitro Prospect and In Vivo Therapeutic Efficacy for the Treatment of Depression

**DOI:** 10.3390/pharmaceutics14030656

**Published:** 2022-03-16

**Authors:** Farheen Fatima Qizilbash, Muhammad Usama Ashhar, Ameeduzzafar Zafar, Zufika Qamar, Annu, Javed Ali, Sanjula Baboota, Mohammed M. Ghoneim, Sultan Alshehri, Asgar Ali

**Affiliations:** 1 Department of Pharmaceutics, School of Pharmaceutical Education and Research, Jamia Hamdard University, New Delhi 110062, India; farheenfqizilbash_sch@jamiahamdard.ac.in (F.F.Q.); usamas132@gmail.com (M.U.A.); zufikakaqamar_sch@jamiahamdard.ac.in (Z.Q.); atriannu407@gmail.com (A.); jali@jamiahamdard.ac.in (J.A.); sbaboota@jamiahamdard.ac.in (S.B.); 2 Department of Pharmaceutics, College of Pharmacy, Jouf University, Sakaka 72341, Al-Jouf, Saudi Arabia; zzafarpharmacian@gmail.com or; 3 Department of Pharmacy Practice, College of Pharmacy, AlMaarefa University, Ad Diriyah 13713, Ad Diriyah, Saudi Arabia; mghoneim@mcst.edu.sa; 4 Department of Pharmaceutics, College of Pharmacy, King Saud University, Riyadh 11451, Riyadh, Saudi Arabia; salshehri1@ksu.edu.sa

**Keywords:** naringenin, nanostructured lipid carrier, intranasal delivery, central composite rotatable design, depression

## Abstract

In the current research, a thymoquinone-enriched naringenin (NGN)-loaded nanostructured lipid carrier (NLC) was developed and delivered via the nasal route for depression. Thymoquinone (TQ) oil was used as the liquid lipid and provided synergistic effects. A TQ- and NGN-enriched NLC was developed via the ultrasonication technique and optimized using a central composite rotatable design (CCRD). The optimized NLC exhibited the following properties: droplet size, 84.17 to 86.71 nm; PDI, 0.258 to 0.271; zeta potential, −8.15 to −8.21 mV; and % EE, 87.58 to 88.21%. The in vitro drug release profile showed the supremacy of the TQ-NGN-NLC in comparison to the NGN suspension, with a cumulative drug release of 82.42 ± 1.88% from the NLC and 38.20 ± 0.82% from the drug suspension. Ex vivo permeation study displayed a 2.21-fold increase in nasal permeation of NGN from the NLC compared to the NGN suspension. DPPH study showed the better antioxidant potential of the TQ-NGN-NLC in comparison to NGN alone due to the synergistic effect of NGN and TQ oil. CLSM images revealed deeper permeation of the NGN-NLC (39.9 µm) through the nasal mucosa in comparison to the NGN suspension (20 µm). Pharmacodynamic studies, such as the forced swim test and the locomotor activity test, were assessed in the depressed rat model, which revealed the remarkable antidepressant effect of the TQ-NGN-NLC in comparison to the NGN suspension and the marketed formulation. The results signify the potential of the TQ-enriched NGN-NLC in enhancing brain delivery and the therapeutic effect of NGN for depression treatment.

## 1. Introduction

Depression is a major public health issue that affects all age groups globally. As per the World Health Organization (WHO), depression affects around 280 million people across the globe and is known to be the main factor contributing to global disability [1]. An imbalance in the levels of neurotransmitters (NTs) such as norepinephrine, dopamine, and serotonin, which have a major function that plays a significant role in conducting information through the presynaptic to the postsynaptic neuron, is commonly attributed to the cause of depression [2]. Issues such as oxidative stress, mitochondrial dysfunction, and inflammation are the most explored aspects that occur in patients with depression [3]. Conventionally available treatment for depression is associated with several adverse effects, such as cognitive impairment, tachycardia, etc., most of which have bioavailability issues and thus low therapeutic efficacy. Natural drugs have been widely used in treating depression and anxiety in recent years due to better therapeutic windows and fewer adverse effects. Naringenin (NGN) is a natural flavonoid that is used for treating numerous neurological ailments. It works by hindering monoamine oxidase-A (MAO-A), which results in the restoration of serotonin, epinephrine, and dopamine levels [4]. It also raises the levels of brain-derived neurotrophic factor (BDNF) in the hippocampus, demonstrating its effectiveness in treating depression [5,6]. NGN, when administered by conventional routes, leads to extensive first-pass metabolism with reduced bioavailability of 5.81% in the brain because of its inability to pass via the blood/brain barrier (BBB) [7]. Therefore, TQ-enriched NLC has been used to achieve a synergistic effect and enhance the BA of NGN in the brain.

Nanostructured lipid carriers (NLCs) were developed because they offer several benefits, including drug protection, low toxicity, no organic solvents during manufacture, and controlled release [8,9]. NLCs also protect the loaded medication from degradation and efflux ions, leading to increased drug bioavailability in the blood and brain [10]. NLCs can also be administered via the intranasal route to achieve brain-targeted action. Since the target site for depression is the brain, the administration of an NLC via the nasal route can result in direct delivery of the medication into the brain by avoiding drug delivery to nontarget sites. The intranasal route is known to circumvent the hepatic metabolism and intestinal metabolism, as well as the BBB. It is a non-delivery-invasive and safer route to achieve improved bioavailability and therapeutic effectiveness of a drug with minimal side effects [11,12].

As one of the components of NLCs is a liquid lipid, thymoquinone (TQ) has been employed as an oil phase or a liquid lipid for the development of NLCs. Thymoquinone (TQ) is known to be the chief component of *Nigella sativa* seeds and has several pharmacological actions, such as anti-inflammatory and antioxidant effects [13]. It also offers peroxidation to the lipid membrane, hindering neuroinflammation by blocking the development of inflammatory mediators, known to be a causative factor for the progression of depression [14,15]. TQ also helps in modulating γ-aminobutyric acid (GABA) and nitric oxide levels and upregulates the levels of 5-hydroxytryptamine in the brain, showing its antidepressant activity [16,17]. Thus, using TQ as a liquid lipid in the formulation provides an added advantage in the treatment of depression [18].

Natural products have been demonstrated to have considerable pharmacological effects that affect many important cell signaling pathways and induce mitogenic, cytotoxic, and genotoxic reactions needed for disease treatment and prophylaxis [19,20]. These herbs, including various flavonoids, triterpenoids, and saponins, have been explored by 75% of the world for various ailments. Some work by hindering the monoamine oxidase, serotonin, dopamine, and norepinephrine transporters, while others work by enhancing hippocampal dopamine levels. Some traditional Chinese medicinal plants, such as flavonoids extracted from *Tilia Americana*, have been found to be effective in treating sleep disorders, while some plants, such as *Polygala tenuifolia, Panax ginseng, Fructus aurantii*, and Shen Yuan, are medication extensively used to stimulate BDNF in depressive models of rodents [21]. Herbal extracts are known to target various biological pathways and several receptors [22]. However, the basic mechanisms of these herbs have been repeatedly described without a fundamental pathway. Thus, these herbs and their extracts need to be further explored in terms of clinical studies to understand their underlying pathways and therapeutic efficacy in humans [23,24].

In recent years, scientists have discovered the use of chemicals, such as statins, chemically known as 3-hydroxy-3-methyl-glutaryl-CoA (HMG-CoA) inhibitors, derived from plants, which have gained attention due to their therapeutic effects that occur by targeting several molecular pathways. These statins, including fluvastatin, simvastatin, atorvastatin, and pravastatin, are known to have significant effects in lowering cholesterol levels, while lovastatin, derived from *Aspergillus terreus*, was approved by the Food and Drug Administration in 1987 to stimulate autophagy, which further suppresses the growth of cancer cells [25,26].

As a matter of fact, some anticancer drugs, such as vinca alkaloids and paclitaxel, have been made from plant sources and used as first-line treatment for ovarian cancer [27]. Another compound, known as apigenin, has gained attention due to its pharmacological effects, such as anti-inflammation and neuroprotective effects. It helps in targeting several signaling pathways, such as phosphatidylinositol 3-kinase/protein kinase B (PI3K/AKT), nuclear factor kappa B (NF-kB), etc., and thus helps in the treatment of cancer by diminishing the proliferation of tumors [28]. Celastrol is another compound also known to possess an anticancer effect by stimulating various cellular pathways, which further works by triggering the mitochondrial apoptotic pathway and hindering NF-kB, resulting in cell cycle arrest. It is also known to tackle bioavailability issues when loaded into a nanocarrier and has proved to be a safer compound. These phytochemicals have expanded their use due to their safer toxicity windows and multifactorial effects [29].

From previous studies, the neurodegenerative effects of NGN and TQ have been established separately against Alzheimer’s disease, Parkinson’s diseases, depression, anxiety, etc. However, there are various limitations associated with NGN, such as low aqueous solubility, poor absorption across the gastrointestinal tract, and extensive gut flora metabolism, leading to the deterioration of NGN [30]. Similarly, TQ also suffers from low bioavailability due to its chemical properties and poor penetration ability across the membrane [31]. Thus, to mask these associated limitations of NGN and TQ, this combination was loaded into an NLC for the treatment of depression and was administered through the intranasal route for uninterrupted delivery to the brain.

Previously, Gaba et al. reported an NGN nanoemulsion encapsulated with vitamin E for treating Parkinson’s disease [32]. The nanoemulsion approach requires the incorporation of large quantities of surfactants and cosurfactants that can be reduced by formulating them into an NLC. Similarly, Ahmad et al. reported a poloxamer/chitosan-loaded NGN nanoemulsion for the treatment of cerebral ischemia [33]. However, both studies lacked a discussion of the stability issues of the prepared nanoemulsion. According to a study, nanoemulsions exhibit major stability issues such as coalescence, Ostwald ripening, creaming, phase separation, burst release [34], and sedimentation upon storage or during the preparation of the formulation [35]. However, these issues can be circumvented by incorporating the drug into an NLC-based formulation. Alam et al. prepared a TQ-loaded solid lipid carrier to attain an antidepressant effect in Wistar rats [36]. This study did not include in vitro and ex vivo assessment of the prepared formulation, which is a major pharmaceutical consideration for assessing the drug release pattern of a formulation. However, the drug loading efficiency in a solid lipid nanocarrier is comparatively lower than that of NLCs. Single lipids are incorporated into solid lipid nanocarriers due to their limited drug loading ability. Additionally, solid lipid nanocarriers undergo gelation and polymorphic transitions due to their highly ordered crystal lattice structure; therefore, NLCs may be a better approach compared to other nanolipid carriers [37].

The purpose of this research work was to formulate and evaluate a naringenin-encapsulated nanostructured lipid carrier (NGN-NLC) with TQ oil as a liquid lipid to provide synergistic efficacy for treating depression. This study also focuses on enhancing the central nervous system (CNS) bioavailability of NGN by avoiding the BBB via the intranasal route. The antidepressant potential of the NGN-NLC was further investigated by performing pharmacodynamic studies.

## 2. Materials and Methods

### 2.1. Materials

Naringenin and TQ oil were purchased as gift samples from Sigma-Aldrich Co. (Spruce St Saint Louis, MO, USA). Gelucire 39/01, glyceryl monostearate, Compritol, Precirol ATO-5, Labrafil M 2130 CS, and Cremophor-EL were purchased from Gattefosse India Pvt. Ltd (Mumbai, India). Ascorbic acid and Rhodamine B were purchased from Sigma-Aldrich (Mumbai, India). Methanol and ethanol were obtained from Merck (Mumbai, India). 1,1-Diphenyl-2-picrylhydrazyl and 5,5′-dithiobis-2-nitrobenzoic acid were purchased from Sigma-Aldrich Chemicals Pvt. Ltd. (Bangalore, Karnataka, India). Sefsol^®^ 218 was purchased from S D Fine Chem Limited (Mumbai, India). All other purchased solvents and chemicals were of analytical grade.

### 2.2. Methods

#### 2.2.1. Solid Lipid and Liquid Lipid Screening

The selection of the solid lipid was made on the basis of the solubility of a drug in the lipid. A 1 g amount of several solid lipids was added to separate vials and heated on a magnetic stirrer at 5 ± 1 °C, taken a few degrees beyond the melting point of the solid lipid. Then, the addition of NGN in an incremental order was performed until saturation was reached, which was assessed visibly. The solubility of NGN in the liquid lipid, which was taken as TQ oil, was assessed by the addition of an excessive quantity of NGN to 2 mL of TQ oil and Sefsol^®^ 218 (S-218) in the ratio of 1:1 in a stoppered vial with a 5 mL capacity [38]. Thereafter, for 5 min, the mixture was vortexed and placed in an isothermal shaker for 48 h at a temperature of 25 ± 0.5 °C. The supernatant obtained was later collected, diluted by methanol, and examined by UV spectrophotometry (UV-1601, Shimadzu, Japan) at 287 nm that was done after performing the centrifugation which was carried out for 13 min at 3000 rpm [39].

#### 2.2.2. Assessment of Binary Mixture

The binary mixture of liquid lipid (TQ oil:S-218) and solid lipid was taken in several ratios (90:10, 85:15, 80:20, 70:30, 75:25, and 60:40) to assess the miscibility between the two lipids. The lipid mixture contained Precirol ATO-5 as the solid lipid, and TQ oil along with S-218 (1:1) as liquid lipids were taken in the above-mentioned ratios and stirred at 200 rpm on a magnetic stirrer for 1 h maintained at 85 ± 1 °C. A cooled sample of the solid combination was smeared on filter paper to evaluate the miscibility, followed by a visual inspection of the filter paper for the occurrence of any liquid oil droplets. The ratio that did not show any appearance of droplets of the lipids on the filter paper was preferred for the development of the NGN-NLC [40,41].

#### 2.2.3. Screening of Surfactants

The surfactant was preferred based on its emulsification capability to emulsify the lipidic mixture. Different surfactants were chosen based on the literature review and their capacity to liquefy lipids. Methylene chloride (3 mL) was employed to liquefy the 100 mg lipidic mixture (Precirol ATO-5 and TQ oil/S-218 in a 1:1 ratio), which was added in the ratio of 70:30 to 10 mL of 5% solution of various surfactants. The organic phase was heated at 40 ± 1 °C for 30 min to obtain a mixture free from methylene chloride. The diluted sample’s percentage transmittance was evaluated using a UV spectrophotometer at 510 nm [42].

### 2.3. Optimization and Formulation of Nanostructured Lipid Carrier (NLC)

#### 2.3.1. NLC Optimization

The prepared NLC was optimized using Design Expert^®^ (version 12.0.1.0, State-Ease Inc. Minneapolis, MN, USA). The binary lipid phase and surfactant were taken as independent variables in the central composite rotatable design (CCRD), and droplet size, entrapment efficiency (% EE), and polydispersity index (PDI) were taken as dependent variables (Table 1).

#### 2.3.2. Formulation of NLC

The solvent diffusion method was used to formulate the NGN-NLC, followed by the ultrasonication method. The preparation of the organic phase was done by prepending the lipidic mixture (70:30) and half the quantity of the surfactant (Cremophor-EL), along with NGN (0.72 mg/mL). Whereas, the preparation of aqueous phase was carried out by adding the left over quantity of the surfactant to 10 mL of distilled water. Both phases were maintained at the same temperature (70 °C), with continuous stirring at 500 rpm on a magnetic stirrer. Dropwise addition of the aqueous phase to the organic phase on a magnetic stirrer was performed with uninterrupted stirring at 800 rpm for 30 min, keeping both phases at 70 ± 5 °C. The resultant was then sonicated for 5 min using a probe sonicator (Hielscher, Germany) in an ice bath, that was subsequently cooled to room temperature [42].

### 2.4. Characterization of Optimized NGN-NLC Formulation

#### 2.4.1. Zeta Potential, Droplet Size, and Polydispersity Index (PDI)

Zeta potential, droplet size, and PDI were assessed using the technique of dynamic light scattering (Malvern Zetasizer, Nano ZS, UK). The optimized NGN-NLC was diluted approximately 50 times before the analysis of the above-mentioned parameters. The scattering angle was maintained at 90°, whereas the temperature was maintained around 25 ± 2 °C. For the formulations that were not diluted, distilled water was used as a blank before experimenting. Experiments were performed in triplicate [43,44].

#### 2.4.2. Entrapment Efficiency (% EE)

The quantity of free drug in the NLC that was entrapped was calculated by estimating the % EE. A 1 mL volume of the NGN-NLC was centrifuged at 4 ± 1 °C for 1 h at 15,120 g force (Sigma-3K30, Osterode am Harz, Germany). After diluting with methanol, the supernatant was collected, and the free drug was analyzed using UV spectroscopy at 287 nm. The calculation of the % EE was performed using the following equation [45].

(1) $$Entrapment\;Efficiency=\frac{\left(W_{t}-W_{s}\right)}{\left(W_{t}\right)}\times 100$$

where, *W_t_* is the quantity of NGN added to the system, and *W_s_* is the quantity of NGN in the supernatant upon centrifugation.

#### 2.4.3. Determination of Surface Morphology

TEM (CM 200, Philips Briarcliff Manor, NY, USA)) was implemented to determine the surface morphology of the optimized NGN-NLC (CM 200, Philips Briarcliff Manor, NY, USA)). The optimized NGN-NLC formulation was subjected to dilution following the addition of a drop of the NGN-NLC on a copper grid coated with carbon, which was then dried and negatively stained with 1% phosphotungstic acid. The grid was then placed in the instrument and examined by TEM [46].

#### 2.4.4. Stability Studies

Stability studies of the NGN-NLC were carried out for 3 months at room temperature (25 ± 2 °C/60 ± 5% RH). The samples of these studies were taken at 0, 1, and 3 months and later examined for variation in physical appearance, droplet size, PDI, and % EE [47].

#### 2.4.5. Drug Release Studies Using Dialysis Membrane

The release study of the drug from the NGN-NLC and NGN suspension were performed on a magnetic stirrer with the aid of a dialysis membrane with assembly placed at 37 ± 2 °C. In the dialysis bag (12–14 KD), 2 mL of the optimized NGN-NLC formulation and NGN suspension containing 0.72 mg/mL of NGN was placed, which was kept in phosphate-buffered solution (PBS, pH = 6.4) (100 mL). A 1 mL volume of the sample was taken at 0.25, 0.5, 1, 2, 4, and 12 h and substituted with the same quantity of fresh media (PBS, pH = 6.4). The samples were examined three times (*n* = 3) by UV spectroscopy at 287 nm [48]. Then, drug release by the dialysis membrane was calculated using the following equation:

(2) $$\%\;Drug\;Release=\frac{Concentration\;(\mu g/\mathrm{mL})\times Dilution\;Factor\times Volume\;of\;Release\;Medium\;\left(\mathrm{mL}\right)}{Initial\;Dose\;\left(\mu g\right)}\times 100\;$$

#### 2.4.6. Ex Vivo Nasal Permeation Study

The depth permeation of the NGN-NLC and NGN-suspension across goat nasal mucosa was evaluated using the CLSM technique, in which the nasal mucosa was positioned on a Franz diffusion cell filled with PBS (pH = 6.4). The NGN-NLC and NGN suspension treated with Rhodamine B (0.03% *w*/*v*) were added to the donor compartment of the Franz diffusion cell. The whole setup was placed on a magnetic stirrer, which was stirred at 100 rpm and maintained at 36 °C for 2 h. The experiment was carried out using isolated goat nasal mucosa and not an artificial membrane, as it was not easily available from a nearby slaughterhouse. Moreover, it was derived from the literature review that goat nasal mucosa is readily and cheaply available, and its morphology is relatively similar to that of humans. A 1 mL volume of the optimized NGN-NLC and NGN suspension both containing 0.72 mg/mL of NGN was placed in the donor section, the volume of which was 10 mL. A 1 mL volume of the sample was taken at 0.25, 0.5, 1, 2, 4, and 12 h and replaced with the same quantity of fresh media (PBS, pH = 6.4). The samples were analyzed in triplicate (*n* = 3) employing UV spectroscopy at 287 nm. The amount of NGN permeated was estimated using the following equation [49]:

(3) $$Permeability\;Coefficient\;\left(P_{app}\right)=\frac{Flux}{Initial\;Drug\;Concentration}$$

#### 2.4.7. Antioxidant Activity: DPPH Assay

Dithiobis-2-nitrobenzoic acid (DPPH) that is known to be a stable radical that produces a deep violet color upon the delocalization of its spare electron or hydrogen radical. The antioxidant potential of the NGN-NLC was compared to a standard of ascorbic acid, as well as pure NGN suspension. The evaluation was based on the ability of DPPH to scavenge free radicals at room temperature. Various concentrations (1–80 µg/mL) of all three samples were prepared in methanol, and each sample (1 mL) was diluted by the methanolic solution of DPPH (1 mL). After 30 min, the mixture was scanned, maintaining methanol (95%) as a blank at 515 nm [50]. The percentage inhibition of DPPH was determined using the following equation:

(4) $$\%\;Inhibition\;of\;DPPH\;Radical=\frac{A_{0}-A_{1}}{A_{0}}\times 100$$

where *A*_0_ and *A*_1_ are the absorbance of the control (blank) and sample, respectively. The 50% inhibitory dose, i.e., IC_50_ value, was evaluated using GraphPad Prism 8.0 (GraphPad Software, San Diego, CA, USA).

#### 2.4.8. Estimation of Depth of Permeation by Confocal Laser Scanning Microscopy (CLSM)

The depth of permeation of the formulation and suspension across goat nasal mucosa was evaluated using the CLSM technique, in which the nasal mucosa was employed on a Franz diffusion cell filled with PBS (pH = 6.4). Then, the nasal mucosa was removed after 2 h, washed using distilled water, and isolated at a temperature of −20 °C, followed by cutting off approximately 20 µm of thin slices of isolated nasal mucosa kept on coverslips made of glass. The formulation and suspension were treated with Rhodamine B (0.03% *w*/*v*), which was added to the donor compartment of the Franz diffusion cell. The whole setup was placed on a magnetic stirrer, which was stirred at 100 rpm and maintained at 36 °C for 2 h. These sections were later assessed on an LSM 710 scanning confocal microscope (TCS SP5II, Leica Microsystem Ltd., Wetzlar, Germany) at 580 nm, where the extent of depth of penetration between the NGN-NLC and NGN suspension was compared [51,52].

### 2.5. Pharmacodynamic Studies

The therapeutic effectiveness of the antidepressant drugs was investigated by performing behavior studies. NTs present in the brain are responsible for the normal functioning of the brain. Thus, the current research work was performed to examine the effect of the NGN-NLC (i.n) when compared to that of the NGN suspension (i.n) and oral administration of the duloxetine suspension (marketed formulation) by performing a forced swim test and locomotor activity test. The formulations were evaluated for inducing their antidepressant effect on rats to downregulate the symptoms linked with depression.

Wistar rats weighing between 200 and 250 g (11–12 weeks old) of either sex were chosen for performing pharmacodynamic tests. The animal study protocol was permitted by the Institutional Animal Ethical Committee (IAEC) (Jamia Hamdard, New Delhi, India), with the approved animal study protocol 173/Go/Re/ S/2000/CPCSEA (Approval No. 1647, 2019). The current study was performed following the guidelines of the Declaration of Helsinki. The experiment was conducted in a manner to reduce suffering. The Wistar rats were categorized into five groups, with three rats in each group. Normal and control groups consisted of non-depressed and depressed rats, respectively. The DLX suspension (oral) (duloxetine suspension) group consisted of depressed rats that were treated with a marketed antidepressant, i.e., DLX suspension with a dosage of 2.06 mg kg^−1^. The doses of the different formulations were administered before determining the pharmacodynamic parameters.

Depressed rats of the last two groups were NGN suspension (i.n) and NGN-NLC (i.n) (0.72 mg kg^−1^). Forced swim tests and locomotor activity tests (behavioral studies) were performed on the above-mentioned groups.

To induce depression, Wistar rats weighing between 200 and 250 g were chosen for the experiment. The rats were positioned in a cylindrical glass tank individually containing water up to a depth of approximately 30 cm at 28 ± 2 °C for 15 min. The animals were then taken out of the water and dried gently by patting them with a clean and dry towel. This procedure was performed for 15 days, and on the same day (15th day), pharmacodynamic parameters were determined after performing the forced swim test and the locomotor activity test [53].

#### 2.5.1. Forced Swim Test

The forced swim test relies on the phenomenon of causing immobility in rats, which is subsequently tested for reversal by antidepressant drug delivery. Swimming time, immobility time, and climbing time are the three components used in the forced swim test. This test is based on the stimulation of immobility. It is a fast, easy, common, and cost-effective test, where immobility represents depressive behavior in rats. The rats were laid in a cylinder-shaped tank with a depth of 30 cm consisting of water at an optimal temperature of 28 ± 2 °C. The 6 min test was performed 30 min after administration of each formulation in the respective groups for a period of 15 days. Animals were re-exposed to a similar environment to swim for 6 min after each dose was administered on the 15th day, and forced swimming, immobility, and climbing times were recorded for 300 s [54].

#### 2.5.2. Locomotor Activity Test

The locomotor activity test is also employed to investigate the efficacy of antidepressant drugs. The locomotor test was conducted to calculate the movements of rats using a digital photoactometer (Hicon, Chandigarh, India) consisting of photocells of infrared light. Rats were positioned in the photoactometer for 15 days before the experiment. The number of times the animal moved was counted using beam light, which was considered the locomotor activity performed by the rats. Different group comparisons were performed after the administration of the NGN-NLC and suspensions [55].

### 2.6. Statistical Analysis

The outcomes of the studies were analyzed by GraphPad Prism 8.0 (GraphPad Software, San Diego, CA, USA). The studies were carried out three times, and the results were expressed as the mean ± standard deviation (SD) using one-way ANOVA analysis. Data were considered statistically significant at *p* < 0.05.

## 3. Results and Discussion

### 3.1. Selection of Liquid Lipid and Solid Lipid

TQ oil was chosen as the liquid lipid for the formulation of the NLC due to its therapeutic effect as a potent antidepressant. In depression, an imbalance of brain NTs such as dopamine and serotonin occurs. TQ oil has been reported to restore these brain chemicals, thus indicating its antidepressant property. TQ oil also exhibits enhanced resistance to oxidative stress by diminishing the elevated levels of superoxide dismutase, lipid peroxidase, malondialdehyde, and glutathione in the brain and has been shown to reduce the pro-inflammatory mediators responsible for depression [56,57]. Thus, TQ oil was chosen as the lipidic phase. The drug solubility (NGN) in TQ oil was found to be 11.43 ± 0.09 mg/mL. This concentration of the selected liquid lipid led to the deposition of the drug at the bottom. Therefore, to enhance the solubility of NGN in TQ oil, S-218 was added as a solubilizing agent. The result was obtained after the incorporation of TQ oil with S-218 in the ratio of 1:1, indicating increased solubility of the drug in the selected liquid lipid. The solubility thus obtained was found to be 16.32 ± 0.21 mg/mL. Hence TQ: S-218 in the ratio of 1:1 was selected [58].

**Figure 1:**
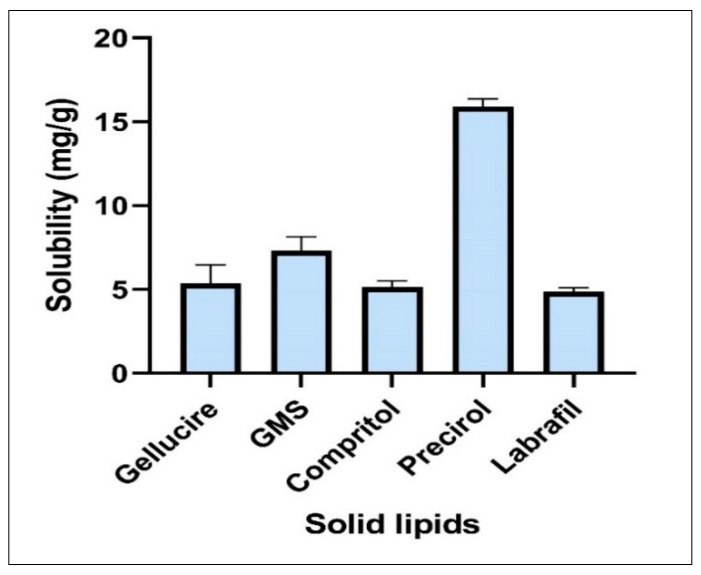
Solubility of NGN in various solid lipids: Precirol ATO-5 exhibited maximum solubility = 16.32 ± 0.21 mg/mL. Data expressed using mean ± SD (*n* = 3).

Among the various solid lipids, Gelucire 39/01, glyceryl monostearate (GMS), Compritol, Precirol ATO-5, and Labrafil M 2130 CS were screened. The highest solubility of NGN was in Precirol ATO-5, which was 15.91 ± 0.47 mg/g, as shown in Figure 1. On mixing with liquid lipid, Precirol ATO-5 has been reported to offer drug release in a sustained manner, improve solubility, and lessen the adverse effects associated with the drug [58,59].

### 3.2. Assessment of Binary Mixture

The optimized ratio of binary lipid was chosen based on the stability of the binary lipid. On performing the test on the filter paper, it was perceived that no oil spots were detected on it after the application of a smear of the binary lipids consisting of a ratio of 70:30, signifying the miscibility of both lipids (i.e., solid and liquid lipids) compared to the other ratios. Hence, Precirol ATO-5 and TQ oil/S-218 (1:1) in the ratio of 70:30 were chosen for the formulation [56].

### 3.3. Screening of Surfactants

Various surfactants were employed to emulsify the selected binary mixture. The percentage transmittance of dispersion of the selected ratio was determined with different surfactants (Table 2). It was found that the drug demonstrated maximum miscibility in Cremophor-EL, including the utmost percent transmittance with the binary mixture; thus, it was considered a surfactant for the development of the NLC. Cremophor-EL is a surfactant used for poorly water-soluble drugs and also provides stability to the NGN-NLC by linking the long-chain fatty acids into the core of the lipidic phase [56].

### 3.4. Optimization and Formulation of Nanostructured Lipid Carrier (NLC)

A total of 13 runs were generated by the CCRD based on the selected independent variables. According to the suggested runs (Table 3), preparation of various formulations was performed and considered to predict the outcome of the dependent variables on the independent variables (*R*1, *R*2, and *R*3). Adjusted *R*2 and predicted *R*2 values of all dependents are mentioned in Table 4.

#### 3.4.1. Outcome of the Independent Variables on Dependent Variables

Outcome of binary mixture concentration and surfactant concentration on the dependent variable (droplet size).

**Figure 2:**
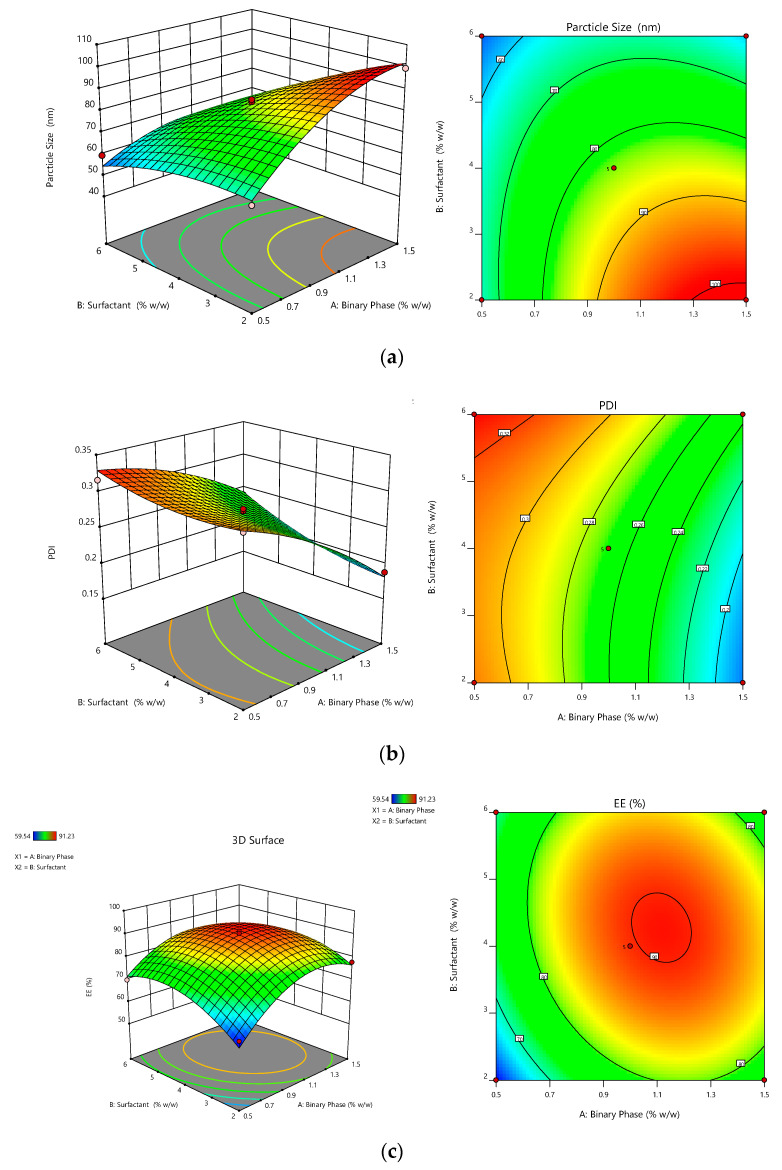
Response surface graphs showing effect of binary mixture concentration and surfactant concentration. (**a**) Droplet size: concentration of binary mixture increased with an increase in droplet size, while concentration of surfactant increased with an increase in droplet size. (**b**) PDI: concentration of binary mixture decreased with an increase in PDI, while with an increase in concentration of surfactant, PDI increased. (**c**) % EE: concentration of binary mixture increased with an increase in % EE, while the concentration of surfactant also increased with an increase in % EE.

The concentration of surfactant had a significant effect on the size of the droplet (*p* < 0.0001), followed by the concentration of the binary lipidic phase (*p* < 0.0002). The size of the droplet of the respective batch was found to be in the range of 49.32–99.39 nm. The binary lipid phase had a positive effect on droplet size, while surfactant concentration had a negative effect, according to the quadratic equation. This also suggested that the binary phase concentration increased as the droplet size of the formulation increased. The effect of the concentration of surfactant on droplet size was such that increasing surfactant concentration decreased droplet size. This was attributed to a decline in the interfacial tension between the oil and aqueous phases, which resulted in a smaller droplet size [57]. The combination of independent variables such as binary lipid and surfactant concentration (AB) had a negative effect on droplet size (Figure 2a).

*R*1 = 83.37 + 10.45A − 13.17B − 7.68AB − 8.83A^2^ − 4.06B^2^ (5)

The outcome of binary mixture concentration and surfactant concentration on the dependent variable (PDI).

The PDI is one of the most crucial factors in developing the NGN-NLC. The concentration of surfactant had a significant effect on the size of the droplet (*p* < 0.0001), succeeded by the concentration of the binary lipidic phase (*p* < 0.0001). On applying analysis, the experimental data were analyzed and fitted in the quadratic model. The PDI of the respective batch was in the range of 0.162–0.326. The quadratic equation showed that the concentration of surfactant had a positive effect, whereas the concentration of the binary lipid phase had a negative effect on the PDI. However, the effect was not that significant. The effect of the various factors on the PDI is shown in Figure 2b.

*R*2 = + 0.26190 – 0.0537A + 0.0202B + 0.0110AB – 0.0142A^2^ + 0.0116B^2^ (6)

The outcome of binary mixture concentration and surfactant concentration on the dependent variable (% EE).

% EE had a main role in the formulation of the NGN-NLC. It was witnessed that the concentration of the binary lipid phase (*p* < 0.0005) and surfactant (*p* < 0.0138) showed a significant effect on the entrapment efficiency. The prepared NGN-NLC showed % EE in the range of 59.54–91.23%. The quadratic equation showed that the binary lipid and the surfactant concentration had a positive effect. This suggested that the entrapment efficiency of the formulation increased with an increase in the concentration of the binary lipid, as well as the concentration of surfactant. Combining the two lipids may result in an improved amalgamation of the drug into the binary lipid, followed by the solubility of the drug and assimilation into voids of the imperfect binary lipid matrix. The multilayered presence of the molecules around the droplets enabled more space for NGN to be incorporated, causing increased entrapment efficiency [58]. On the other hand, the combination of the independent variables such as the binary lipid and surfactant concentration (AB) displayed a negative effect on the entrapment efficiency. The effect of different factors on entrapment efficiency is shown in Figure 2c.

*R*3 = +89.69 + 5.44 + 2.91B − 2.97AB − 10.79A^2^ − 7.95B^2^ (7)

#### 3.4.2. Validation of Experimental Design

A comparison was made between the validation of the experimental design for the optimized formulation, and the obtained responses revealed an optimal formula consisting of 1% binary lipid mixture concentration and 4% concentration of surfactant. The experimental results were found to be analogous with each other denoting the validation and reliability for the optimized formulation.

### 3.5. Evaluation of Optimized NGN-NLC

#### 3.5.1. Zeta Potential, Droplet Size, and Polydispersity Index (PDI)

**Figure 3:**
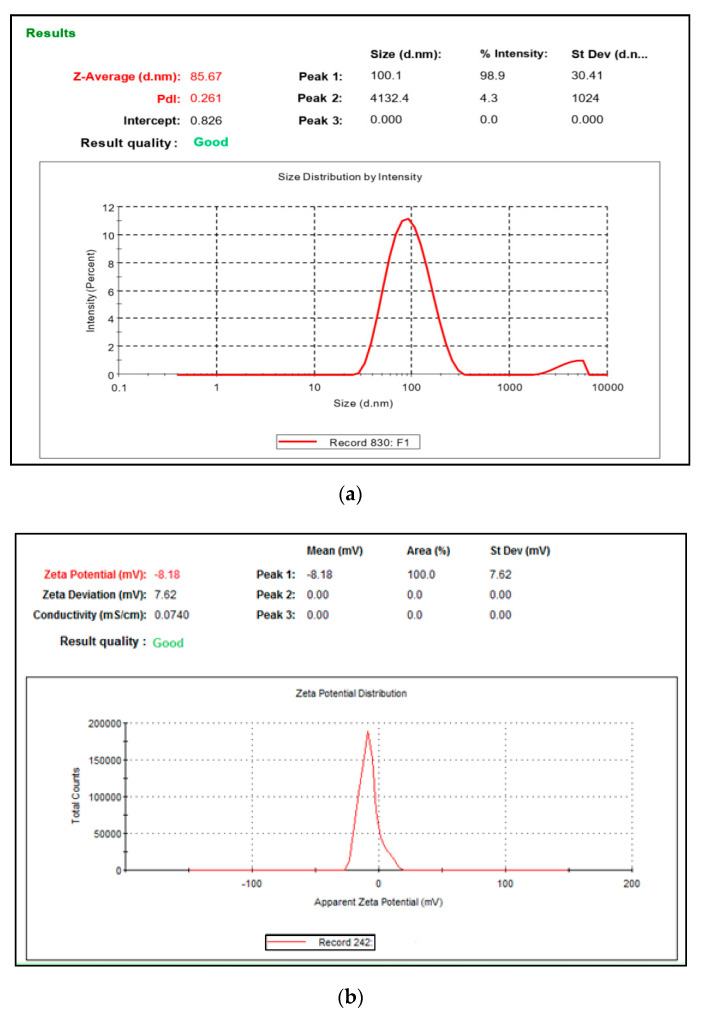
(**a**) Zeta potential of optimized NGN-NLC was found to be in the range of −8.15 to −8.21 mV. (**b**) Droplet size and PDI of optimized NGN-NLC were found to be in the range of 84.17 to 86.71 nm and 0.258 to 0.271, respectively.

The value of zeta potential of the optimized NGN-NLC was in the range of −8.15 to −8.21 mV (Figure 3a). Here, the negative charge could be credited to the lipids and nonionic surfactant used in the formulation, leading to a lower magnitude of nano-based particles without the presence of aggregation, thus indicating the stability of the NGN-NLC [59].

The droplet size and PDI of the optimized NGN-NLC were found to be in the range of 84.17 to 86.71 nm and 0.258 to 0.271, respectively (Figure 3b). The droplet size of the formulations has a significant role in the absorption of drugs via the i.n route because of the opening and closing of the tight junctions located in the epithelium cells of the nasal membrane. Smaller droplet size and larger surface area would help in the rapid absorption of the drug. Furthermore, a decreased size of the droplet would result in improved drug carriage via the olfactory route to the brain. The PDI signifies the distribution of the size of the nanocarriers in the formulation. PDI values less than 0.5 signify that the formulation is monodispersed (homogeneity), whereas a PDI value more than 0.5 signifies the polydispersity of the formulation [60].

#### 3.5.2. Entrapment Efficiency (% EE)

The optimized formulation exhibited a range of 87.58 to 88.21% for % EE, which signified that the drug was entrapped into the nanocarrier-mediated system. The lipophilicity of NGN could be the reason for the high entrapment efficiency that allowed it to solubilize in the lipid matrix. NLCs have irregularities in the crystal lattice that enable adequate space for drug molecules to be accommodated, further resulting in enhanced drug entrapment efficiency. The main goal of the greater % EE is to attain a maximal drug concentration in the lipidic matrix that results in a greater amount of drug release at a lower dose [61].

#### 3.5.3. Determination of Surface Morphology

**Figure 4:**
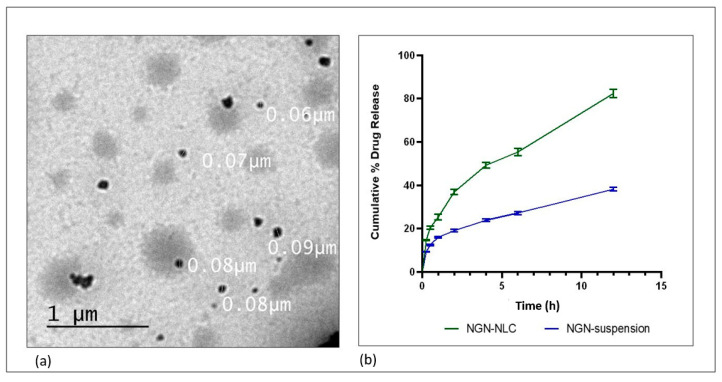
(**a**) Surface morphology determination using TEM, showing the uniform distribution of droplets in optimized NGN-NLC. (**b**) The drug release profile of NGN from NGN-NLC and NGN suspension in phosphate buffer (pH = 6.4) using the dialysis membrane, showed a 2.15-fold increase in drug release from NGN-NLC in comparison to that of NGN suspension. The result was found to be statistically significant, whereas data were expressed using mean ± SD (*n* = 3).

TEM was employed to determine the morphology of the surface of the optimized formulation. The droplets of the NGN-NLC seemed to be dark and spherical with uniform droplet size distribution with a diameter of 60–90 nm (0.06–0.09 µm) (Figure 4a). No aggregation of particles was observed in the formulation.

### 3.6. Stability Studies

The 3-month stability studies confirmed the stability of the NGN-NLC at room temperature (25 ± 2 °C/60 ± 5% RH). The NGN-NLC was assessed for changes in physical appearance, occurrence of phase separation, caking, changes in droplet size, PDI, and entrapment efficiency. The NGN-NLC did not show any changes in physical appearance phase separation and caking. An insignificant change (*p* > 0.05) was observed in the droplet size, PDI, and % EE of the formulation. The formulation did not show any precipitate formation (Table 5). The high stability of the formulation could be due to the high zeta potential and small droplet size [62].

### 3.7. Drug Release Studies by Dialysis Membrane

The drug release study was performed using a dialysis membrane to investigate the release of NGN from the NGN-NLC and NGN suspension in phosphate buffer (pH = 6.4), respectively. The percentage cumulative release of NGN from the formulation was found to be 82.42 ± 1.88% in 12 h; however, in the case of the NGN suspension, the release of the drug was way too slow, with only 38.20 ± 0.82% drug release after 12 h along with drug precipitation in the dialysis bag. Initially, the drug was seen to escape quickly from the lipidic surface of the NGN-NLC, followed by a slower release pattern due to drug release from the lipid matrix. The results were found to be statistically significant (*p* > 0.001). The optimized NGN-NLC displayed the biphasic type of release from the NGN-NLC and NGN suspension. The NGN-NLC had a 12 h release time (Figure 4b), which could be due to the lower droplet size of the generated NLC.

The results signified that the drug release of the NGN-NLC followed the Korsmeyer–Peppas release model because of the maximum value of the coefficient of correlation (*R*2), i.e., 0.9958, and followed the Fickian diffusion with a biphasic drug release pattern, thus proving the encapsulation of the drug in the core of the lipid. The NGN-NLC showed a 2.15-fold increase in drug release compared to that of the NGN suspension, thereby proving its effectiveness in drug release studies.

### 3.8. Ex Vivo Nasal Permeation Study

**Figure 5:**
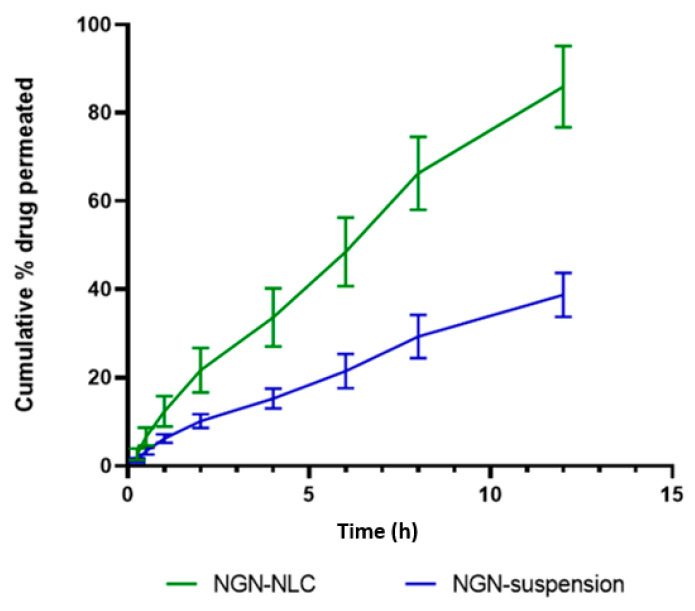
Ex vivo permeation of NGN from the NGN-NLC and NGN-suspension on isolated goat nasal mucosa, showing 2.21-fold increase in NGN from NGN-NLC in comparison to NGN suspension. Data expressed using mean ± SD (*n* = 3).

The ex vivo permeation study was used to compare the permeation of the drug (NGN) from the NLC and suspension by means of goat nasal mucosa as a diffusion barrier on the Franz diffusion cell. The NGN-NLC revealed 85.91 ± 9.23% permeation in 12 h in contrast to the NGN suspension, which revealed 38.76 ± 5.00% permeation (Figure 5). The steady-state flux for the NGN-NLC and NGN suspension was found to be 17.7 µgcm^−2^ h^−1^ and 1.56 10^−1^ µgcm^−2^ h^−1^, respectively, while the permeability coefficient was found to be 8.54 µgcm^−2^ h^−1^ and 0.74 10^−1^ µgcm^−2^ h^−1^ for the NGN-NLC and NGN suspension, respectively. The flux and permeability coefficient of the NGN-NLC was comparatively higher than that of the NGN suspension. The results were found to be statistically significant (*p* > 0.001). The pattern of drug release from the ex vivo permeation studies was seen to be in correspondence with that of the in vitro studies. The NGN-NLC displayed a 2.21-fold increase in drug permeation compared to that of the NGN suspension. This could be due to the higher retention time of the NGN-NLC, which further led to an enhanced permeation rate and also to the desired sustained release of the drugs across the nasal mucosa that acted as a reservoir for drugs, thereby proving its effectiveness in drug permeation studies [63].

### 3.9. Antioxidant Activity: DPPH Assay

**Figure 6:**
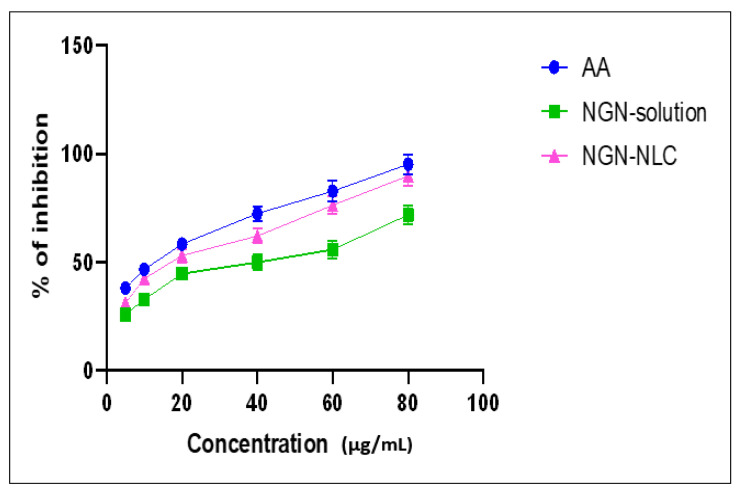
Antioxidant potential of AA, NGN solution, and NGN-NLC, showing % inhibition increase of 1.2-fold from NGN-NLC in comparison with NGN solution due to synergistic effect of NGN and TQ. Data expressed using mean ± SD (*n* = 3).

The reducing potential of DPPH was assessed by observing the change in color from purple to yellow which was evaluated at absorbance 515 nm. The data displayed that free radical scavenging was strongly influenced by the NGN-NLC. The increase in the antioxidant property of nanoformulations is usually preferred for improving their potential in neurological brain disorders [50]. The antioxidant activity of the NGN-NLC was found to be higher because of the combined effect of NGN and TQ, thus proving their efficacy as strong antioxidants (Figure 6). It is predicted that the reason for effective antioxidant potential in the NGN-NLC could be due to the synergistic effect of NGN along with TQ oil possessing powerful antioxidant activity. The IC_50_ value for the NGN solution, NGN-NLC, and ascorbic acid was found to be 41.79 μg/mL, 23 μg/mL, and 14 μg/mL, respectively [63].

### 3.10. Estimation of Depth of Permeation by Confocal Laser Scanning Microscopy (CLSM)

**Figure 7:**
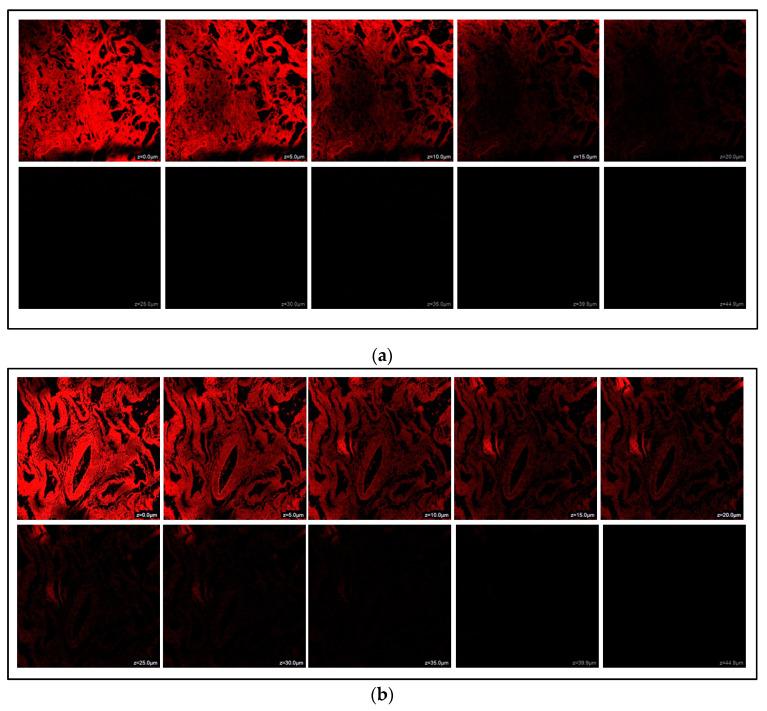
(**a**) CLSM images after administration of NGN suspension across isolated goat nasal mucosa. (**b**) CLSM images after administration of NGN-NLC across isolated goat nasal mucosa, showing 1.99-fold enhanced permeation in comparison to NGN suspension.

CLSM investigation was conducted to assess the permeability of the NGN-NLC and NGN suspension across the goat’s nasal mucosa. It was observed that the maximum intensity of fluorescence for the NGN suspension was up to a depth of 20.0 µm, which diminished at 25.0 µm (Figure 7a). However, for the NGN-NLC, it was spotted up to a depth of 39.9 µm, that diminished at 44.9 µm (Figure 7b), indicating the uniform distribution of NGN in the NGN-NLC compared to that of the NGN suspension. Thus, the NGN-NLC displayed about 1.99-fold deeper permeation in the nasal mucosa in comparison with the NGN suspension. These results were found to be in accordance with the ex vivo permeation study. 

### 3.11. Pharmacodynamic Studies

#### 3.11.1. Forced Swim Test (FST)

The forced swim test is the generally used animal model for evaluating the effectiveness of antidepressants. The FST revealed that the control group showed a mean swimming time of 82.33 ± 1.42 s. Additionally, on the administration of the NGN suspension via the i.n route, a significant increase (*p* < 0.001) in the mean swimming time (162.66 ± 2.22 s) was observed compared to the DLX suspension after following the oral administration, which showed a mean swimming time of 131.33 ± 1.56 s.

**Figure 8:**
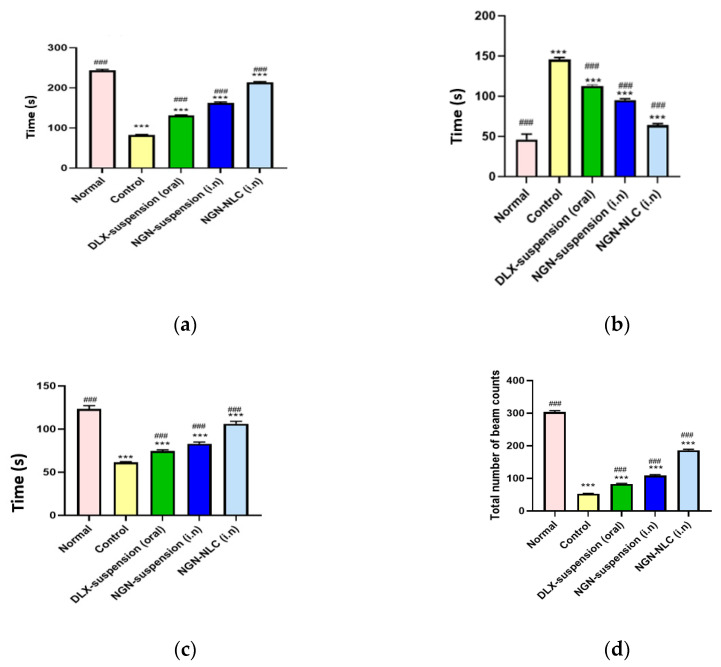
Pharmacodynamic investigation of different groups after performing behavioral studies such as (**a**) forced swim test, (**b**) immobility test, (**c**) climbing time, and (**d**) locomotor activity test. (*** indicates that the comparison was made between normal vs. other groups; whereas ### indicates that the comparison was made between control and other groups). Data expressed using mean ± SD (*n* = 3).

However, after the administration of the NGN-NLC via the i.n route, a significant increase (*p* < 0.001 versus control) with a mean swimming time of 213 ± 2.51 s indicated the therapeutic effectiveness of the formulation (Figure 8a).

The immobility time refers to the motionless behavior of rats when they were placed in a cylinder consisting of water. The mean immobility time of the control group showed a significant increase (*p* < 0.001) compared to that of the DLX suspension (oral) group. After the administration of the NGN suspension through the i.n route, a significant decrease (*p* < 0.001) was observed in contrast to the control group. On the other hand, the administration of the NGN-NLC (i.n) displayed a significant decrease (*p* < 0.001) in the immobility time (64 ± 1.52 s) against that of the control group (Figure 8b).

The climbing time for the normal and control groups was found to be 123.33 ± 3.78 and 61.33 ± 3.87 s, respectively. The depressed group treated with the DLX suspension orally and the NGN suspension intranasally showed a climbing time of 74.66 ± 1.21 and 83.01 ± 1.89 s, respectively. The depressed group treated with the NGN-NLC via the i.n route showed a climbing time of about 106 ± 3.03 s (Figure 8c). It was observed that the rats exhibited a fall in the time spent trying to escape from the cylindrical glass tank filled with water, but this time duration was significantly extended after the administration of the NGN-NLC (*p* < 0.001) via the i.n route against the control group, the DLX suspension (oral) group and the group treated with the NGN suspension (i.n). The significant increase in the climbing time of rats treated with the NGN-NLC (i.n) could be credited to the occurrence of NGN and TQ oil in the hippocampus of the brain, leading to the synergistic effect of the drugs, and thus allowing the inhibition of the reuptake of serotonin, thus causing an enhanced amount of serotonin in the brain [64].

#### 3.11.2. Locomotor Activity Test

The locomotor activity test was conducted with the help of a digital photoactometer for 5 min. It was observed that the normal and control groups showed a beam count of 304 ± 3.51 and 52.66 ± 1.12 s, respectively, while the DLX suspension (oral) and NGN suspension (i.n) groups were seen to exhibit a total beam count of 83.33 ± 1.52 and 109.01 ± 2.64 s (*p* < 0.001). However, after the administration of the NGN-NLC (i.n), a significant increase of *p* < 0.001 with a total beam count of 186.33 ± 2.82 s against that of the control group was witnessed (Figure 8d). The results proved the efficacy of the NGN-NLC when administered via the i.n route in comparison to that of the NGN suspension and DLX suspension, which were administered intranasally and orally, respectively [65].

Successful outcomes were obtained from the pharmacodynamic studies consisting of the FST and the locomotor activity test, which further proved the therapeutic efficacy and synergistic effect after the i.n administration of the NGN-NLC. The administration of the NGN-NLC not only enhanced the uptake of drugs through the i.n route against suspensions but also led to the desired antidepressant effect. The therapeutic effectiveness of the NGN-NLC (i.n) could be attributed to the lipid nanocarriers that helped in the direct targeting of the brain through the olfactory route, whereas the orally administered formulation showed poor results because of the extensive hepatic first-pass metabolism and its inability to cross via tight junctions present at the BBB. Nonetheless, the mechanisms exhibited by the NTs led to behavioral changes exhibited by the rats. The increase in the concentration of serotonin in the hypothalamus and frontal cortex of rats was attributed to the i.n administration of the NGN-NLC, which further increased the availability of serotonin in the presynaptic neurons and caused a remarkable increase in the mean swimming and climbing times of the rats [64,65].

## 4. Conclusions

The NGN-NLC was successfully prepared and optimized utilizing a CCRD. The optimized NLC exhibited the following properties: droplet size, 84.17 to 86.71 nm; polydispersity index, 0.258 to 0.271; zeta potential, −8.15 to −8.21 mV; and entrapment efficiency, 87.58 to 88.21%. The NGN-NLC showed controlled release for 12 h that revealed 2.15-fold enhancement of NGN release from the NLC compared to the NGN suspension. Ex vivo results showed enhanced permeation (2.21-fold) of the NLC through the nasal mucosa compared to the NGN suspension. DPPH study revealed the improved antioxidant potential of NGN. The results of the CLSM study showed a higher amount of penetration by the NGN-NLC than the NGN suspension, while the stability study showed good stability of the NLC over 3 months. Furthermore, pharmacodynamic data revealed an increase in the antidepressant potential of the NGN-NLC. The present study concluded the successful optimization of the NGN-NLC and its potential in augmenting brain bioavailability and the therapeutic effectiveness of NGN for the effective treatment of depression after nasal administration. The research findings indicated that transporting the drug via the olfactory pathway can help in targeting the drug directly to the brain. Nevertheless, the current research work still lacks clinical study data, which can help in assessing its therapeutic effectiveness, as well as the risk/benefit ratio in humans.

## Figures and Tables

**Table 1:** Independent and dependent variables that have been considered in CCRD.

Factors	Levels Used				
Independent Variable	Axial (−α)	Low (−1)	Medium (0)	High (+1)	Axial (+α)
**A**: Binary lipid concentration (*w*/*w*%)	0.292	0.5	1	1.5	1.707
**B**: Surfactant concentration (*w*/*w*%)	1.171	2	4	6	6.828
**Dependent variable**	Constraints used				
***R*1**: Droplet size (nm)	Minimum				
***R*2**: PDI	Minimum				
***R*3**: Entrapment efficiency (%)	Maximum				

**Table 2:** Transmittance in various surfactants (*n* = 3).

Surfactants	Transmittance (%) ± S.D
Tween 80	40.61 ± 0.53
Tween 20	70.18 ± 0.09
Cremophor-EL	91.11 ± 0.67
Labrasol	0.40 ± 0.35
Poloxamer	41.20 ± 0.77
Tween 60	25.36 ± 0.55
Span 20	0.40 ± 0.73
Solutol	0.73

**Table 3:** CCRD experimental design and observed responses.

Runs	Independent Variable		Dependent Variable		
	Factor 1 A: Binary Mixture Concentration (Solid/Liquid Lipid Concentration) (*w*/*w*)	Factor 2 B: Surfactant Concentration (*w*/*w*)	*R*1: Droplet Size (nm)	*R*2: PDI	*R*3: (% EE)
1	0.29	4.0	49.32	0.326	59.54
2	0.50	6.0	59.43	0.316	69.88
3	0.50	2.0	63.43	0.309	62.34
4	1.00	1.1	97.44	0.259	66.78
5	1.00	4.0	82.33	0.261	88.99
6	1.00	6.8	50.32	0.332	80.98
7	1.00	4.0	81.88	0.277	91.23
8	1.00	4.0	84.34	0.267	89.45
9	1.00	4.0	82.65	0.274	88.43
10	1.00	4.0	85.32	0.266	90.34
11	1.50	6.0	64.66	0.239	73.42
12	1.50	2.0	99.39	0.188	77.78
13	1.70	4.0	79.33	0.162	76.87

**Table 4:** Overall ANOVA analysis results for CCRD.

Response	Statistics of Model Summary				Suggested Model
	Std. Dev.	*R*2	Adjusted *R*2	Predicted *R*2	
***R*1: Droplet Size**	4.14	0.9628	0.9362	0.7500	Quadratic
***R*2: PDI**	0.0087	0.9824	0.9698	0.8818	Quadratic
***R*3: EE%**	2.52	0.9702	0.9490	0.8067	Quadratic

**Table 5:** Stability studies of NGN-NLC at room temperature (25 ± 2 °C/60 ± 5% RH) (*n* = 3).

Time (Months)	Change in Appearance	Phase Separation	Caking	Droplet Size (nm) (Mean ± SD) (*n* = 3)	PDI (Mean ± SD) (*n* = 3)	% EE (Mean ± SD) (*n* = 3)
0	No	No	No	85.43 ± 1.21	0.263 ± 0.012	87.58 ± 4.69
1	No	No	No	85.96 ± 1.59	0.271 ± 0.015	87.21 ± 3.46
3	No	No	No	86.21 ± 3.90	0.281 ± 0.017	86.57 ± 3.55

## Data Availability

This study did not report any data (All the data is included in the current manuscript).

## Abbreviations

AA: ascorbic acid, BBB; blood/brain barrier, CCRD; central composite rotatable design, CLSM; confocal laser scanning microscopy, CPCSEA; Committee for the Purpose of Control and Supervision of Experiments on Animals, DLX suspension; duloxetine suspension, DPPH; dithiobis-2-nitrobenzoic acid, % EE; entrapment efficiency, i.n; intranasal, MAO; monoamine oxidase, NLC; nanostructured lipid carrier, NGN-NLC; naringenin-loaded nanostructured lipid carrier, NT; neurotransmitter, PDI; polydispersity index, S-218; Sefsol^®^, SD; standard deviation, TEM; transmission electron microscope, TQ; thymoquinone.
